# Supplementary material for: Discovery and application of insertion-deletion (INDEL) polymorphisms for QTL mapping of early life-history traits in Atlantic salmon
Source: BMC Genomics. 2010 Mar 8;11:156. doi: 10.1186/1471-2164-11-156 (PMC2838853; doi:10.1186/1471-2164-11-156)
Supplement: Additional file 2 — Information on developed 76 locus single-run INDEL panel in Atlantic salmon. Information on fluorescence labeling, primer concentrations, PCR pooling and links to alignments, INDEL motifs and GENESCAN (Burge and Karlin 1997) predictions of genes/exons are available in html format. [file 1471-2164-11-156-S2.ZIP › Additionalfile2/table467.html]

Contig Alignment

```
Base	A B C D E F   
1	. . G . . .    
2	. . G G . .    
3	. . G G . .    
4	. . G G . .    
5	. . G G . .    
6	. . A A . .    
7	. . G G . .    
8	. . A A . .    
9	. . C C . .    
10	. . A A . .    
11	. . A A . .    
12	. . C C . .    
13	. . A A . .    
14	. . A A . .    
15	. . C C . .    
16	. . A A . .    
17	. . T T . .    
18	. . G G . .    
19	. . G G . .    
20	C . C C . .    
21	G . G G . .    
22	T . T T . .    
23	C . C C . .    
24	G . G G . .    
25	A . A A . .    
26	G . G G . .    
27	G . G G . .    
28	G . G G . .    
29	C C C C . .    
30	A A A A . .    
31	C C C C . .    
32	C C C C . .    
33	A A A A . .    
34	G G G G . .    
35	C C C C . .    
36	A A A A . .    
37	A A A A . .    
38	C C C C . .    
39	T T T T . .    
40	A A A A . .    
41	C C C C . .    
42	C C C C . .    
43	G G G G . .    
44	G G G G . .    
45	C C C C . .    
46	A A A A . .    
47	G G G G . .    
48	A A A A . .    
49	C C C C . .    
50	T T T T . .    
51	C C C C . .    
52	C C C C . .    
53	T T T T . .    
54	G G G G . .    
55	G G G G . .    
56	T T T T . .    
57	C C C C . .    
58	A A A A . .    
59	G G G G . .    
60	C C C C . .    
61	T T T T . .    
62	T T T T . .    
63	T T T T . .    
64	C C C C . .    
65	G G G G . .    
66	T T T T . .    
67	A A A A . .    
68	A A A A . .    
69	A A A A . .    
70	T T T T . .    
71	G G G G . .    
72	G G G G . .    
73	T T T T . .    
74	A A A A . .    
75	C C C C . .    
76	T T T T . .    
77	A A A A . .    
78	C C C C . .    
79	A A A A . .    
80	A A A A . .    
81	T T T T . .    
82	G G G G . .    
83	C C C C . .    
84	T T T T . .    
85	G G G G . .    
86	C C C C . .    
87	T T T T . .    
88	G G G G . .    
89	G G G G . .    
90	A A A A . .    
91	T T T T . .    
92	T T T T . .    
93	C C C C . .    
94	A A A A . .    
95	A A A A . .    
96	C C C C . .    
97	A A A A . .    
98	A A A A . .    
99	A A A A . .    
100	A A A A . .    
101	T T T T . .    
102	T T T T . .    
103	G G G G . .    
104	G G G G . .    
105	T T T T . .    
106	C C C C . .    
107	T T T T . .    
108	G G G G . .    
109	A A A A . .    
110	T T T T . .    
111	G G G G . .    
112	C C C C . .    
113	G G G G . .    
114	T T T T . .    
115	G G G G . .    
116	A A A G . .    
117	T T T T . .    
118	G G G G . .    
119	A A A A . .    
120	C C C C . .    
121	A A A A . .    
122	C C C C . .    
123	A A A A . .    
124	A A A A . .    
125	T T T T . .    
126	C C C C . .    
127	C C C C . .    
128	A A A A . .    
129	T T T T . .    
130	G G G G . .    
131	A A A A . .    
132	G G G G . .    
133	G G G G . .    
134	A A A A . .    
135	T T T T . .    
136	A A A A . .    
137	G G G G . .    
138	C C C C . .    
139	G G G G . .    
140	A A A A . .    
141	C C C C . .    
142	G G G G . .    
143	T T T T . .    
144	A A A A . .    
145	A A A A . .    
146	A A A A . .    
147	A A A A . .    
148	G G G G . .    
149	A A A A . .    
150	G G G G . .    
151	G G G G . .    
152	C C C C . .    
153	C C C C . .    
154	C C C C . .    
155	T T T T . .    
156	G G G G . .    
157	C C C C . .    
158	G G G G . .    
159	G G G G . .    
160	A A A A . .    
161	G G G G . .    
162	G G G G . .    
163	C C C C . .    
164	T T T T . .    
165	C C C C . .    
166	C C C C . .    
167	C C C C . .    
168	A A A A . .    
169	G G G G . .    
170	A A A A . .    
171	G G G G . .    
172	A A A A . .    
173	A A A A . .    
174	G G G G . .    
175	G G G G . .    
176	T T T T . .    
177	G G G G . .    
178	T T T T . .    
179	A A A A . .    
180	C C C C . .    
181	A A A A . .    
182	A A A A . .    
183	C C C C . .    
184	G G G G . .    
185	A A A A . .    
186	C C C C . .    
187	A A A A . .    
188	G G G G . .    
189	G G G G . .    
190	A A A A . .    
191	T T T T . .    
192	G G G G . .    
193	T T T T . .    
194	T T T T . .    
195	C C C C . .    
196	A A A A . .    
197	G G G G . .    
198	G G G G . .    
199	C C C C . .    
200	T T T T . .    
201	C C C C . .    
202	A A A A . .    
203	A A A A . .    
204	G G G G . .    
205	A A A A . .    
206	G G G G . .    
207	A A A A . .    
208	G G G G . .    
209	C C C C . .    
210	C C C C . .    
211	C C C C . .    
212	T T T T . .    
213	G G G G . .    
214	G G G G . .    
215	A A A A . .    
216	C C C C . .    
217	C C C C . .    
218	T T T T . .    
219	C C C C . .    
220	T T T T . .    
221	C C C C . .    
222	C C C C . .    
223	A A A A . .    
224	T T T T . .    
225	G G G G . .    
226	A A A A . .    
227	A A A A . .    
228	G G G G . .    
229	C C C C . .    
230	A A A A . .    
231	G G G G . .    
232	G G G G . .    
233	C C C C . .    
234	G G G G . .    
235	G G G G . .    
236	T T T T . .    
237	T T T T . .    
238	C C C C . .    
239	T T T T . .    
240	G G G G . .    
241	C C C C . .    
242	C C C C . .    
243	C C C C . .    
244	A A A A . .    
245	A A A A . .    
246	G G G G . .    
247	G G G G . .    
248	A A A A . .    
249	A A A A . .    
250	C C C C . .    
251	A A A A . .    
252	G G G G . .    
253	C C C C . .    
254	G G G G . .    
255	G G G G . .    
256	A A A A . .    
257	C C C C . .    
258	T T T T . .    
259	C C C C . .    
260	A A A A . .    
261	A A A A . .    
262	T T T T . .    
263	A A A A . .    
264	C C C C . .    
265	G G G G . .    
266	A A A A . .    
267	G G G G . .    
268	G G G G . .    
269	A A A A . .    
270	G G G G . .    
271	G G G G . .    
272	T T T T . .    
273	A A A A . .    
274	C C C C . .    
275	A A A A . .    
276	G G G G . .    
277	C C C C . .    
278	A A A A . .    
279	G G G G . .    
280	A A A A . .    
281	C C C C . .    
282	C C C C . .    
283	T T T T . .    
284	G G G G . .    
285	G G G G . .    
286	G G G G . .    
287	C C C C . .    
288	T T T T . .    
289	C C C C . .    
290	A A A A . .    
291	A A A A . .    
292	A A A A . .    
293	T T T T . .    
294	G G G G . .    
295	T T T T . .    
296	C C C C . .    
297	G G G G . .    
298	T T T T . .    
299	T T T T . .    
300	T T T T . .    
301	A A A A . .    
302	A A A A . .    
303	T T T T . .    
304	G G G G . .    
305	G G G G . .    
306	A A A A . .    
307	C C C C . .    
308	T T T T . .    
309	A A A A . .    
310	A A A A . .    
311	T T T T . .    
312	A A A A . .    
313	A A A A . .    
314	A A A A . .    
315	A A A A . .    
316	A A A A . .    
317	G G G G . .    
318	T T T T . .    
319	C C C C . .    
320	C C C C . .    
321	T T T T . .    
322	A A A A . .    
323	A A A A . .    
324	A A A A . .    
325	A A A A . .    
326	C C C C . .    
327	T T T T . .    
328	G G G G . .    
329	C C C C . .    
330	A A A A . .    
331	A A A A . .    
332	A A A A . .    
333	C C C C . .    
334	T T T T . .    
335	C C C C . .    
336	C C C C . .    
337	A A A A . .    
338	C C C C . .    
339	T T T T . .    
340	C C C C . .    
341	A A A A . .    
342	T T T T . .    
343	C C C C . .    
344	T T T T . .    
345	G G G G . .    
346	G G G G . .    
347	C C C C . .    
348	A A A A . .    
349	C C C C . .    
350	T T T T . .    
351	C C C C . .    
352	C C C C . C    
353	A A A A . A    
354	A A A A . A    
355	G G G G . G    
356	C C C C . C    
357	A A A A . A    
358	G G G G . G    
359	G G G G . G    
360	C C C C . C    
361	T T T T . T    
362	A A A A . A    
363	G G G G . G    
364	C C C C . C    
365	G G G G . G    
366	C C C C . C    
367	A A A A . A    
368	A A A A . A    
369	A A A A . A    
370	C C C C . C    
371	G G G G . G    
372	C C C C . C    
373	T T T T . T    
374	G G G G . G    
375	A A A A . A    
376	A A A A . A    
377	G G G G . G    
378	T T T T . T    
379	A A A A . A    
380	T T T T . T    
381	T T T T . T    
382	T T T T . T    
383	G G G G . G    
384	A A A A . A    
385	A A A A . A    
386	A A A A . A    
387	T T T T . T    
388	A A A A . A    
389	A A A A . A    
390	T T T T . T    
391	T T T T . T    
392	C C C C . C    
393	A A A A . A    
394	A A A A . A    
395	A A A A . A    
396	T T T T . T    
397	A A A A . A    
398	A A A A . A    
399	T T T T . T    
400	A A A A . A    
401	T T T T . T    
402	T T T T . T    
403	T T T T . T    
404	G G G G . G    
405	A A A A . A    
406	A A A A . A    
407	C C C C . C    
408	C C C C . C    
409	C C C C . C    
410	A A A A . A    
411	G G G G . G    
412	G G G G . G    
413	T T T T . T    
414	C C C C . C    
415	T T T T . T    
416	G G G G . G    
417	A A A A . A    
418	G G G G . G    
419	G G G G . G    
420	T T T T . T    
421	A A A A . A    
422	C C C C . C    
423	A A A A . A    
424	G G G G . G    
425	T T T T . T    
426	G G G G . G    
427	C C C C . C    
428	A A A A . A    
429	T T T T . T    
430	A A A A . A    
431	T T T T . T    
432	A A A A . A    
433	G G G G . G    
434	T T T T . T    
435	C C C C . C    
436	T T T T . T    
437	C C C C . C    
438	A A A A . A    
439	C C C C . C    
440	A A A A . A    
441	C C - C . -  2	 indel  
442	A A A A . A    
443	T T T T . T    
444	T T T T . T    
445	A A A A . A    
446	G G G G . G    
447	T T T T . T    
448	T T T T . T    
449	T T T T . T    
450	G G G G . G    
451	T T T T . T    
452	A A A A . A    
453	C C C C . C    
454	A A A A . A    
455	G G G G . G    
456	A A A A . A    
457	A A A A . A    
458	A A A A . A    
459	T T T T . T    
460	G G G G . G    
461	G G G G . G    
462	C C C C . C    
463	T T T T . T    
464	T T T T . T    
465	T T T T . T    
466	G G G G . G    
467	A A A A . A    
468	G G G G . G    
469	A A A A . A    
470	T T T T . T    
471	G G G G . G    
472	C C C C . C    
473	C C C C . C    
474	A A A A . A    
475	C C C C . C    
476	T T T T . T    
477	G G G G . G    
478	G G G G . G    
479	G G G G . G    
480	G G G G . G    
481	A A A A . A    
482	A A A A . A    
483	A A A A . A    
484	T T T T . T    
485	G G G G . G    
486	G G G G . G    
487	A A A A . A    
488	A A A A A A    
489	G G G G G G    
490	A A A A A A    
491	T T T T T T    
492	A A A A A A    
493	T T T T T T    
494	G G G G G G    
495	T T T T T T    
496	C C C C C C    
497	C C C C C C    
498	A A A A A A    
499	A A A A A A    
500	A A A A A A    
501	G G G G G G    
502	T T T T T T    
503	T T T T T T    
504	T T - - - -  2	 indel  
505	C C - - - -  2	 indel  
506	A A - - - -  2	 indel  
507	T T - - - -  2	 indel  
508	A A - - - -  2	 indel  
509	G G - - - -  2	 indel  
510	C C - - - -  2	 indel  
511	A A - - - -  2	 indel  
512	G G - - - -  2	 indel  
513	T T - - - -  2	 indel  
514	T T - - - -  2	 indel  
515	G G G G G G    
516	A A A A A A    
517	T T T T T T    
518	G G G G G G    
519	T T T T T T    
520	T T T T T T    
521	A A A A A A    
522	T T T T T T    
523	A A A A A A    
524	G G G G G G    
525	T T T T T T    
526	G G G G G G    
527	A A A A A A    
528	C C C C C C    
529	G G G G G G    
530	A A A A A A    
531	G G G G G G    
532	G G G G G G    
533	T T T T T T    
534	A A A A A A    
535	T T T T T T    
536	T T T T T T    
537	C C C C C C    
538	A A A A A A    
539	A A A A A A    
540	C C C C C C    
541	T T T T T T    
542	C C C C C C    
543	T T T T T T    
544	T T T T T T    
545	A A A A A A    
546	C C C C C C    
547	T T T T T T    
548	C C C C C C    
549	T T T T T T    
550	A A A A A A    
551	C C C C C C    
552	G G G G G G    
553	A A A A A A    
554	G G G G G G    
555	G G G G G G    
556	C C C C C C    
557	C C C C C C    
558	T T T T T T    
559	G G G G G G    
560	G G G G G G    
561	A A A A A A    
562	G G G G G G    
563	T T T T T T    
564	T T T T T T    
565	T T T T T T    
566	T T T T T T    
567	C C C C C C    
568	T T T T T T    
569	G G G G G G    
570	T T T T T T    
571	T T T T T T    
572	C C C C C C    
573	T T T T T T    
574	A A A A A A    
575	C C C T T C  2	 transition  
576	G G G G G G    
577	T T T T T T    
578	G G G G G G    
579	A A A A A A    
580	T T T T T T    
581	A A A A A A    
582	A A A A A A    
583	T T T T T T    
584	T T T T T T    
585	A A A A A A    
586	A A A A A A    
587	T T T T T T    
588	T T T T T T    
589	G G G G G G    
590	C C C C C C    
591	A A A A A A    
592	C C C C C C    
593	C C C C C C    
594	C C C C C C    
595	A A A A A A    
596	C C C C C C    
597	C C C C C C    
598	A A A A A A    
599	G G G G G G    
600	G G G G G G    
601	T T T T T T    
602	G G G G G G    
603	T T T T T T    
604	C C C C C C    
605	C C C C C C    
606	C C C C C C    
607	G G G G G G    
608	G G G G G G    
609	G G G G G G    
610	T T T T T T    
611	C C C C C C    
612	T T T T T T    
613	G G G G G G    
614	A A A A A A    
615	A A A A A A    
616	T T T T T T    
617	C C C C C C    
618	A A A A A A    
619	G G G G G G    
620	T T T T T T    
621	C C C C C C    
622	C C C C C C    
623	C C C C C C    
624	T T T T T T    
625	G G G G G G    
626	A A A A A A    
627	T T T T T T    
628	T T T T T T    
629	A A A A A A    
630	G G G G G G    
631	A A A A A A    
632	G G G G G G    
633	G G G G G G    
634	G G G G G G    
635	G G G G G G    
636	A A A A A A    
637	A A A A A A    
638	C C C C C C    
639	A A A A A A    
640	A A A A A A    
641	T T T T T T    
642	T T T T T T    
643	A A A A A A    
644	A A A A A A    
645	A A A A A A    
646	C C C C C C    
647	G G G G G G    
648	C C C C C C    
649	A A A A A A    
650	A A A A A A    
651	T T T T T T    
652	G G G G G G    
653	G G G G G G    
654	A A A A A A    
655	A A A A A A    
656	C C C C C C    
657	T T T T T T    
658	G G G G G G    
659	G G G G G G    
660	C C C C C C    
661	T T T T T T    
662	T T T T T T    
663	C C C C C C    
664	G G G G G G    
665	A A A A A A    
666	G G G G G G    
667	G G G G G G    
668	T T T T T T    
669	T T T T T T    
670	C C C C C C    
671	G G G G G G    
672	G G G G G G    
673	A A A A A A    
674	G G G G G G    
675	T T T T T T    
676	T T T T T T    
677	G G G G G G    
678	A A A A A A    
679	G G G G G G    
680	G G G G G G    
681	G G G G G G    
682	T T T T T T    
683	T T T T T T    
684	A A A A A A    
685	T T T T T T    
686	A A A A A A    
687	G G G G G G    
688	T T T T T T    
689	G G G G G G    
690	T T T T T T    
691	A A A A A A    
692	A A A A A A    
693	C C C C C C    
694	T T T T T T    
695	T T T T T T    
696	T T T T T T    
697	G G G G G G    
698	G G G G G G    
699	A A A A A A    
700	A A A A A A    
701	T T T T T T    
702	G . G G G G    
703	. . A A A A    
704	. . A A A A    
705	. . G G G G    
706	. . G G G G    
707	. . G G G G    
708	. . C C C C    
709	. . A A A A    
710	. . A A A A    
711	. . A A A A    
712	. . G G G G    
713	. . T T T T    
714	. . C C C C    
715	. . T T T T    
716	. . A A A A    
717	. . G G G G    
718	. . C C C C    
719	. . C C C C    
720	. . T T T T    
721	. . C C C C    
722	. . T T T T    
723	. . G G G G    
724	. . A A A A    
725	. . G G G G    
726	. . G G G G    
727	. . C C C C    
728	. . T T T T    
729	. . A A A A    
730	. . A A A A    
731	. . G G G G    
732	. . G G G G    
733	. . T T T T    
734	. . G G G G    
735	. . G G G G    
736	. . A A A A    
737	. . A A A A    
738	. . A A A A    
739	. . T T T T    
740	. . A A A A    
741	. . T T T T    
742	. . T T T T    
743	. . G G G G    
744	. . T T T T    
745	. . T T T T    
746	. . A A A A    
747	. . T T T T    
748	. . A A A A    
749	. . T T T T    
750	. . T T T T    
751	. . G G G G    
752	. . A A A A    
753	. . T T T T    
754	. . A A A A    
755	. . G G G G    
756	. . T T T T    
757	. . G G G G    
758	. . C C C C    
759	. . T T T T    
760	. . A A A A    
761	. . A A A A    
762	. . A A A A    
763	. . C C C C    
764	. . T T T T    
765	. . A A A A    
766	. . A A A A    
767	. . T T T T    
768	. . T T T T    
769	. . G G G G    
770	. . A A A A    
771	. . A A A A    
772	. . C C C C    
773	. . A A A A    
774	. . A A A A    
775	. . T T T T    
776	. . C C C C    
777	. . A A A A    
778	. . A A A A    
779	. . T T T T    
780	. . G G G G    
781	. . G G G G    
782	. . A A A A    
783	. . G G G G    
784	. . T T T T    
785	. . . A A A    
786	. . . . G G    
787	. . . . G G    
788	. . . . A A    
789	. . . . T T    
790	. . . . A A    
791	. . . . A A    
792	. . . . T T    
793	. . . . T T    
794	. . . . A A    
795	. . . . T T    
796	. . . . G G    
797	. . . . A A    
798	. . . . T T    
799	. . . . T T    
800	. . . . A A    
801	. . . . T T    
802	. . . . G G    
803	. . . . A A    
804	. . . . A A    
805	. . . . G G    
806	. . . . T T    
807	. . . . A A    
808	. . . . T T    
809	. . . . T T    
810	. . . . A A    
811	. . . . G G    
812	. . . . A A    
813	. . . . T T    
814	. . . . T T    
815	. . . . A A    
816	. . . . T T    
817	. . . . G G    
818	. . . . A A    
819	. . . . A A    
820	. . . . G G    
821	. . . . C C    
822	. . . . A A    
823	. . . . A A    
824	. . . . G G    
825	. . . . T T    
826	. . . . A A    
827	. . . . G G    
828	. . . . G G    
829	. . . . A A    
830	. . . . G G    
831	. . . . T T    
832	. . . . A A    
833	. . . . G G    
834	. . . . A A    
835	. . . . G G    
836	. . . . A A    
837	. . . . C C    
838	. . . . T T    
839	. . . . A A    
840	. . . . A A    
841	. . . . A A    
842	. . . . G G    
843	. . . . G G    
844	. . . . T T    
845	. . . . C C    
846	. . . . A A    
847	. . . . G G    
848	. . . . A A    
849	. . . . T T    
850	. . . . T T    
851	. . . . G G    
852	. . . . G G    
853	. . . . A A    
854	. . . . A A    
855	. . . . T T    
856	. . . . T T    
857	. . . . G G    
858	. . . . G G    
859	. . . . G G    
860	. . . . C C    
861	. . . . A A    
862	. . . . A A    
863	. . . . T T    
864	. . . . A A    
865	. . . . G G    
866	. . . . T T    
867	. . . . T T    
868	. . . . G G    
869	. . . . A A    
870	. . . . G G    
871	. . . . T T    
872	. . . . T T    
873	. . . . G G    
874	. . . . G G    
875	. . . . G G    
876	. . . . A A    
877	. . . . G G    
878	. . . . A A    
879	. . . . T T    
880	. . . . G G    
881	. . . . C C    
882	. . . . C C    
883	. . . . T T    
884	. . . . A A    
885	. . . . T T    
886	. . . . A A    
887	. . . . A A    
888	. . . . A A    
889	. . . . G G    
890	. . . . C C    
891	. . . . T T    
892	. . . . G G    
893	. . . . T T    
894	. . . . T T    
895	. . . . T T    
896	. . . . A A    
897	. . . . A A    
898	. . . . T T    
899	. . . . G G    
900	. . . . T T    
901	. . . . T T    
902	. . . . G G    
903	. . . . A A    
904	. . . . A A    
905	. . . . A A    
906	. . . . A A    
907	. . . . A A    
908	. . . . T T    
909	. . . . G G    
910	. . . . A A    
911	. . . . C C    
912	. . . . A A    
913	. . . . T T    
914	. . . . A A    
915	. . . . C C    
916	. . . . G G    
917	. . . . A A    
918	. . . . A A    
919	. . . . A A    
920	. . . . G G    
921	. . . . A A    
922	. . . . T T    
923	. . . . G G    
924	. . . . A A    
925	. . . . C C    
926	. . . . A A    
927	. . . . G G    
928	. . . . A A    
929	. . . . A A    
930	. . . . C C    
931	. . . . T T    
932	. . . . A A    
933	. . . . A A    
934	. . . . T T    
935	. . . . G G    
936	. . . . T T    
937	. . . . T T    
938	. . . . T T    
939	. . . . T T    
940	. . . . G G    
941	. . . . T T    
942	. . . . T T    
943	. . . . T T    
944	. . . . C C    
945	. . . . T T    
946	. . . . G G    
947	. . . . T T    
948	. . . . T T    
949	. . . . T T    
950	. . . . G G    
951	. . . . A A    
952	. . . . G G    
953	. . . . C C    
954	. . . . T T    
955	. . . . T T    
956	. . . . T T    
957	. . . . T T    
958	. . . . T T    
959	. . . . T T    
960	. . . . C C    
961	. . . . T T    
962	. . . . A A    
963	. . . . T T    
964	. . . . A A    
965	. . . . G G    
966	. . . . G G    
967	. . . . A A    
968	. . . . C C    
969	. . . . G G    
970	. . . . T T    
971	. . . . A A    
972	. . . . C C    
973	. . . . A A    
974	. . . . T T    
975	. . . . T T    
976	. . . . A A    
977	. . . . C C    
978	. . . . T T    
979	. . . . T T    
980	. . . . G G    
981	. . . . G G    
982	. . . . A A    
983	. . . . G G    
984	. . . . C C    
985	. . . . C C    
986	. . . . T T    
987	. . . . T T    
988	. . . . A A    
989	. . . . C C    
990	. . . . C C    
991	. . . . T T    
992	. . . . G G    
993	. . . . A A    
994	. . . . A A    
995	. . . . A A    
996	. . . . G G    
997	. . . . A A    
998	. . . . G G    
999	. . . . G G    
1000	. . . . T T    
1001	. . . . C C    
1002	. . . . A A    
1003	. . . . T T    
1004	. . . . C C    
1005	. . . . C C    
1006	. . . . G G    
1007	. . . . T T    
1008	. . . . G G    
1009	. . . . A A    
1010	. . . . G G    
1011	. . . . A A    
1012	. . . . G G    
1013	. . . . G G    
1014	. . . . A A    
1015	. . . . A A    
1016	. . . . A A    
1017	. . . . G G    
1018	. . . . A A    
1019	. . . . G G    
1020	. . . . G G    
1021	. . . . T T    
1022	. . . . T T    
1023	. . . . G G    
1024	. . . . A A    
1025	. . . . G G    
1026	. . . . G G    
1027	. . . . A A    
1028	. . . . G G    
1029	. . . . T T    
1030	. . . . G G    
1031	. . . . G G    
1032	. . . . T T    
1033	. . . . C C    
1034	. . . . G G    
1035	. . . . A A    
1036	. . . . A A    
1037	. . . . G G    
1038	. . . . A A    
1039	. . . . A A    
1040	. . . . A A    
1041	. . . . T T    
1042	. . . . G G    
1043	. . . . A A    
1044	. . . . G G    
1045	. . . . A A    
1046	. . . . T T    
1047	. . . . G G    
1048	. . . . G G    
1049	. . . . A A    
1050	. . . . C C    
1051	. . . . G G    
1052	. . . . T T    
1053	. . . . G G    
1054	. . . . G G    
1055	. . . . A A    
1056	. . . . G G    
1057	. . . . G G    
1058	. . . . C C    
1059	. . . . T T    
1060	. . . . T T    
1061	. . . . G G    
1062	. . . . A A    
1063	. . . . T T    
1064	. . . . G G    
1065	. . . . T T    
1066	. . . . T T    
1067	. . . . C C    
1068	. . . . C C    
1069	. . . . T T    
1070	. . . . T T    
1071	. . . . C C    
1072	. . . . T T    
1073	. . . . C C    
1074	. . . . T T    
1075	. . . . T T    
1076	. . . . T T    
1077	. . . . G G    
1078	. . . . G G    
1079	. . . . T T    
1080	. . . . C C    
1081	. . . . T T    
1082	. . . . G G    
1083	. . . . G G    
1084	. . . . T T    
1085	. . . . G G    
1086	. . . . A A    
1087	. . . . A G    
1088	. . . . . T    
1089	. . . . . G    
1090	. . . . . G    
1091	. . . . . T    
1092	. . . . . T    
1093	. . . . . G    
1094	. . . . . T    
1095	. . . . . C    
1096	. . . . . C    
1097	. . . . . T    
1098	. . . . . C    
1099	. . . . . T    
1100	. . . . . G    
1101	. . . . . T    
1102	. . . . . A    
1103	. . . . . A    
1104	. . . . . T    
1105	. . . . . A    
1106	. . . . . T    
1107	. . . . . T    
1108	. . . . . T    
1109	. . . . . C    
1110	. . . . . A    
1111	. . . . . A    
1112	. . . . . A    
1113	. . . . . T    
1114	. . . . . A    
1115	. . . . . A    
1116	. . . . . A    
1117	. . . . . G    
1118	. . . . . T    
1119	. . . . . G    
1120	. . . . . G    
1121	. . . . . A    
1122	. . . . . C    
1123	. . . . . C    
1124	. . . . . C    
1125	. . . . . T    
1126	. . . . . G    
1127	. . . . . T    
1128	. . . . . G    
1129	. . . . . G    
1130	. . . . . G    
1131	. . . . . A    
1132	. . . . . A    
1133	. . . . . G    
1134	. . . . . A    
1135	. . . . . C    
1136	. . . . . T    
1137	. . . . . G    
1138	. . . . . C    
1139	. . . . . T    
1140	. . . . . T    
1141	. . . . . A    
1142	. . . . . T    
1143	. . . . . T    
1144	. . . . . A    
1145	. . . . . A    
1146	. . . . . A    
1147	. . . . . A    
1148	. . . . . A    
1149	. . . . . T    
1150	. . . . . C    
1151	. . . . . T    
1152	. . . . . A    
1153	. . . . . C    
1154	. . . . . A    
1155	. . . . . T    
1156	. . . . . T    
1157	. . . . . T    
1158	. . . . . T    
1159	. . . . . T    
1160	. . . . . G    
1161	. . . . . G
```
